# Social+Me: a persuasive application to increase communication between students and their support networks in Southern Chile

**DOI:** 10.7717/peerj-cs.848

**Published:** 2022-01-27

**Authors:** Fabián Fernández-Vera, Denisse C. Urrutia, Pedro O. Rossel, Valeria Herskovic, Carolina Fuentes

**Affiliations:** 1 Department of Computer Science, Universidad Católica de la Santísima Concepción, Concepción, Chile; 2 Centro de Investigación en Biodiversidad y Ambientes Sustentables (CIBAS), Universidad Católica de la Santísima Concepción, Concepción, Chile; 3 Department of Computer Science, Pontificia Universidad Católica de Chile, Santiago, Chile; 4 School of Computer Science and Informatics, Cardiff University, Cardiff, United Kingdom

**Keywords:** Persuasive technology, Social network, Mobile, Undergraduate student

## Abstract

Technology can improve university students’ communication, helping them maintain relationships. Although there are many available technological tools, students face challenges—*e.g.,* living far from home, failing grades, depression—that may isolate them from their networks. Most research into these topics has been conducted in countries in which students leave their parents’ home while at university, which is not the case for most students in southern Chile. In this context that has been seldom studied, this paper presents two studies, focusing on two research questions: (1) How do university students in southern Chile communicate? (2) Can a mobile application persuade university students to increase their communication patterns? To answer these questions, we conducted a survey with 90 students in southern Chile, and then developed a persuasive application called Social+Me, aimed at monitoring communication with students’ support networks and persuading them to keep in touch. We conducted a preliminary evaluation of Social+Me, and the application was well received by participants, who felt that it improved their communication with their social network. The main impact of our study lies in applying persuasive technologies to the communicative practice of university students to prevent students from feeling isolated or unsupported.

## Introduction

Young adults use their phones primarily to communicate with others, *e.g.*, through e-mail, social networks, and messaging (Lopez-Fernandez et al., 2017; Hernández-Orellana, Pérez-Garcias & Roco-Videla, 2021). University students feel that technology improves communication and helps them maintain relationships (Russo et al., 2014), using it for academic purposes as well (Russo et al., 2014; Gallardo-Echenique, Bullen & Marqués-Molías, 2016). Although technological tools for communication help students maintain their social ties, students face challenges—*e.g.*, living far from home, failing grades, depression—that may contribute to isolate them from their support networks.

The medium that students use to communicate with their support networks can vary, *e.g.*, as new social networks become more popular, but also, students may use different types of communication according to social tie, *e.g.*, texting with friends but talking with their parents (Russo et al., 2014). Studies have found differences in communication medium use among regions, *e.g.*, (Lopez-Fernandez et al., 2017). Cultural factors affect how students communicate—*e.g.*, a study comparing American and Flemish first-year students found some differences as for Flemish students, transition from high school to university is more gradual than for American students (Abeele & Roe, 2011). The same is true for other countries, such as Chile, in which on-campus student housing is nonexistent and most university students live with their families. However, online communication is still important during the transition to university, and students use it to receive emotional support, *e.g.*, about the challenges in the first stages of university life (Quan-Haase, 2007).

Persuasive technologies are those designed to change a person’s behavior or attitude in some predetermined way (Fogg, 1999). The three most common motivation strategies implemented to persuade users are: tracking and monitoring; audio, visual and textual feedback; and social support, sharing and comparison, though several studies combine multiple strategies (Orji & Moffatt, 2018). The idea behind persuasive technology is to influence positive behaviors, motivating healthy habits and increasing well-being. Persuasive technologies have been used in a variety of domains, *e.g.*, health, gerontechnology, education and environmental conservation (IJsselsteijn et al., 2006).

Australian students may need additional support from friends, parents and families, as few live on-campus (Peel, 2000). Chilean students all live off-campus, so maintaining their social ties, strengthening their communities, and decreasing isolation may be important as well. The objective of this study is therefore twofold: first, to understand how students communicate; and second, to propose a way to increase and give visibility to their communication patterns. This work proposes to apply persuasive technologies to a new domain: university students’ communication practices, which can be encouraged to prevent students from feeling isolated or unsupported.

This paper presents Social+Me, a persuasive application to promote university students’ communication with their closest social networks, that might be used in situations in which users experience isolation. A recent example is the COVID-19 pandemic, which forced education online (Sun, Tang & Zuo, 2020) increasing loneliness and isolation in undergraduate student groups (Hamza et al., 2020; Bu, Steptoe & Fancourt, 2020). Other populations may also experience isolation—*e.g.*, mothers of pediatric cancer patients (Fuentes et al., 2014) and older adults (Nicholson, 2012) in vulnerable settings. We focus on two research questions: ***(1) How do university students in southern Chile communicate?*** ***(2) Can a mobile application persuade university students to increase their communication patterns?***

**Figure 1:**
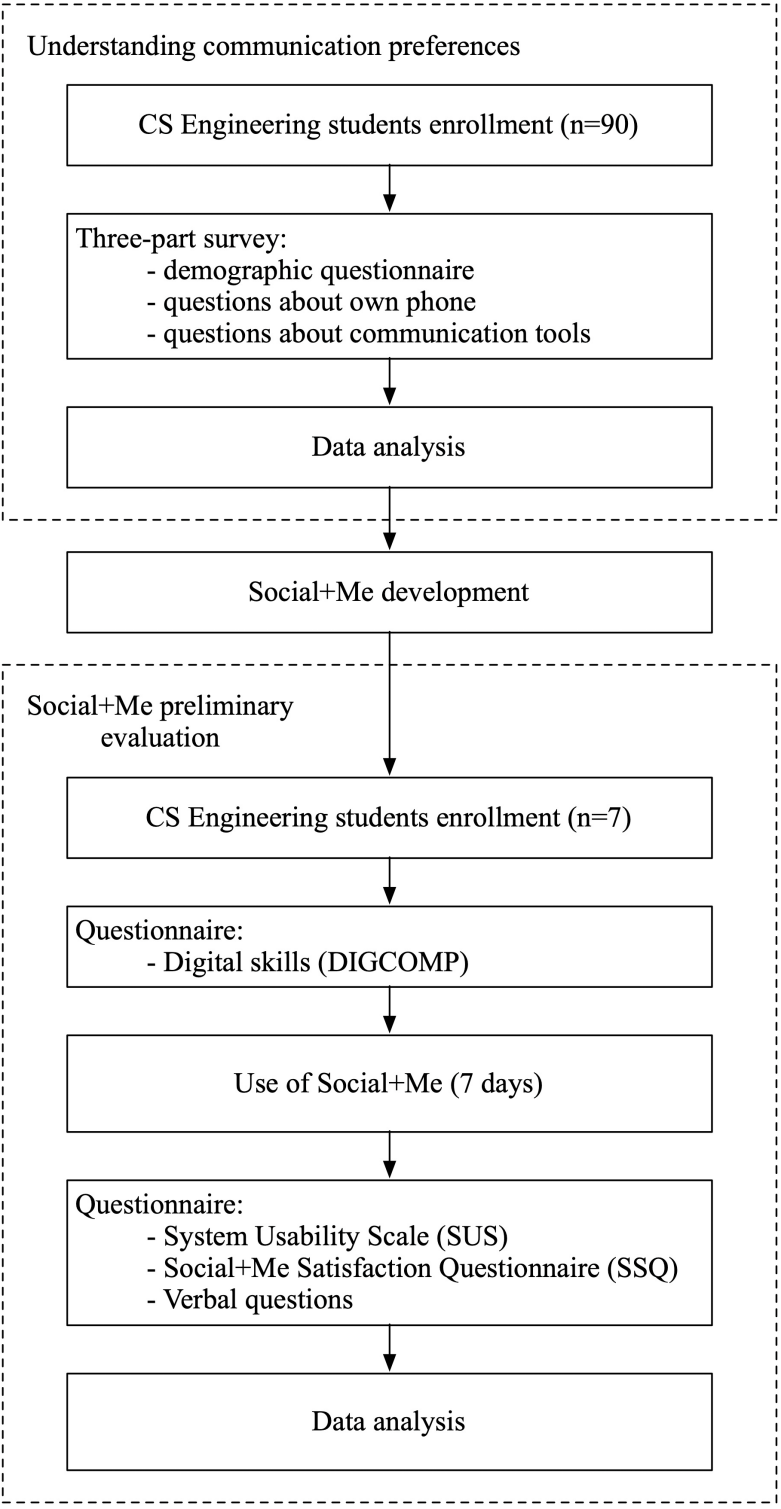
Study process flow.

To answer both research questions, we conducted a three-part study. Initially, to answer the first research question, we conducted a survey to understand university students’ communication preferences. Then, we implemented a persuasive application to monitor smartphone communication, including the communication preferences that students declared to use in the previous phase. Finally, we conducted a preliminary evaluation of the application through a small-scale user study to assess whether users found the application useful and easy to use. We illustrate the process in Fig. 1.

This paper is organized as follows. First, we briefly present contextual information about Chile’s university students and culture. Then, we present related work covering each research question: studies related to university students’ communication preferences, as well as persuasive applications, especially focused on communication. Then, we present the results of the first study, followed by the proposed application, and its evaluation. Finally, we present the conclusion of this work.

## Chilean Context: the University and its Students

In recent years, Chile has increased its Internet access and connectivity. Internet access has increased from 58% in 2013 to 87.8% in 2020–21 (Branch Group, 2021; DataReportal, 2021). Overall, 83.5% of the population uses social networks (Branch Group, 2021; DataReportal, 2021), and Facebook, WhatsApp, YouTube and Instagram are the most popular social networking sites (Fundacin Nativo Digital, 2019). Particularly for young adults aged 18–29, Facebook and WhatsApp are the most used platforms (Gómez-Urrutia & Tello-Navarro, 2020).

Chilean university students commonly live at their parents’ homes during their undergraduate studies. An investigation conducted in 2017 with 60 undergraduate students from three different cities in Chile found that 83% of students live at their parents’ home while in university (Barrera-Herrera & Vinet, 2017). Another study of Chilean university students found that 69% live with their parents, and that most are single, do not have a job (78%) and do not have children (95%) (Barrera-Herrera, Vinet & Ortiz, 2020). Only a small fraction of university students move from their parents’ home, mostly to another city and mainly for academic reasons (Barrera-Herrera & Vinet, 2017).

Within the Chilean culture, social links and family ties have a central role (Gaymer, 2002). Family social support is important to Chilean university students, with special relevance on maintaining their well-being and supporting their mental health (Barrera-Herrera et al., 2019). The core family unit in Chile is the nuclear family, but people often have close relationships to extended family members as well (Cultural Atlas, 2021). These relationships commonly generate close social circles (Gaymer, 2002), in which young adults often stay in the nuclear home until they marry or move for work (Cultural Atlas, 2021). Still, adults will often visit or call their parents regularly (Cultural Atlas, 2021), maintaining close family relationships throughout adulthood.

Similarly to other groups of their age around the world, Chilean undergraduate students communicate using technology with their networks (Gómez-Urrutia & Tello-Navarro, 2020) and their communication preferences are influenced by how important their social links are (Gaymer, 2002). A study conducted in 2021 to understand Chilean university students’ perception of their digital identity revealed that their connectivity habits rely mostly on mobile phones with Internet access, and they spend more than 4 h per day on their phones; it also identifies that *phubbing* practices (paying attention to a mobile phone at the same time that interaction with someone else is happening) are less common when they interact with close family members or friends (Hernández-Orellana, Pérez-Garcias & Roco-Videla, 2021).

## Related Work

### Communication preferences and connection through technology

Young adults spend 3 h a day on average using their smartphones doing activities such as e-mailing, social networking, text messaging, chatting, searching, reading and gaming (Lopez-Fernandez et al., 2017). Social communication has been the main reason students go online, even as early as 2002 (Jones et al., 2009). Previous research has found that university students prefer instant messaging over the phone or face-to-face contact to maintain social ties, using different communication patterns to interact with different contacts (Quan-Haase, 2007; O’Hara et al., 2014). Technology contributes to connecting students and their families: parents and university students rely heavily on technology to maintain contact (Ramsey, Oberhauser & Gentzler, 2015), especially when students live far away from home.

In the past few years, young adults in the US have used social networks such as Facebook, YouTube and Instagram, Tumblr, Snapchat, and Vibe (Lenhart et al., 2010; Villanti et al., 2017; Poushter, Bishop & Chwe, 2018)—for example, in 2021, 95% of adults 18 to 29 use YouTube (Pew Research Center, 2021). There are large generational differences in social media use—*e.g.*, young adults 18 to 24 are much more likely to use Instagram, Snapchat and TikTok than others (Pew Research Center, 2021). In Chile, the most common social networks used by younger adults are Facebook, Twitter, Instagram (Poushter, Bishop & Chwe, 2018), and WhatsApp (Gómez-Urrutia & Tello-Navarro, 2020).

It is not yet clear whether social media and technology helps or hinders social connectedness. Some studies have found that higher social media use correlates to lower social isolation among older adults (Hajek & König, 2019). Conversely, for young adults, higher social media use has been found to correlate with higher perceived social isolation (Primack et al., 2017). Even though connecting through these technologies may not be as beneficial as connecting in person, there are some situations in which personal interaction is not possible. For example, during the COVID-19 pandemic, social isolation impacted university students, who were affected by the lack of personal interaction, as well as decreased motivation, and increased anxiety, boredom and loneliness (Leal Filho et al., 2021).

Few studies on university students’ communication patterns have been previously conducted in Chile, considering particularities such as that university students commonly live at home with their parents during their studies (*e.g.*, 68% of the participants in the current study, while 48% of college students lived at home in the US in 2015 (GoodCall, 2021)) and that no on-campus housing exists. This paper aims to understand communication preferences for these students, and understand whether they can use a persuasive system to help them maintain their social ties.

### Persuasive technology for communication

Persuasive technologies have been developed and studied mainly in two areas: health promotion and disease management, generally focusing on behavior change, attitude, motivation, and awareness. Persuasive technologies have been proposed in other domains as well, *e.g.*, educational settings (Mintz & Aagaard, 2012). Some persuasive technologies have been proposed to support or encourage communication practices, mainly to support older adults’ social interactions through virtual agents acting as companionship, encouraging participation in social activities (Vargheese et al., 2016).

*Gamification* refers to the intentional use of methods inspired by game techniques to engage and influence attention, behaviors and motivation in non-game activities or environments (Krath & von Korflesch, 2021). Gamification is part of persuasive systems, using goals as rewards as part of the persuasive techniques (Krath & von Korflesch, 2021). Gamification has been applied with a focus on communication practices to connect users for social interaction in areas such as sustainability, education and fitness (Krath & von Korflesch, 2021). Using gamification to improve communication skills for social interaction has revealed that people are interested in improving themselves and increasing bonding with friends (Hori et al., 2013; Seaborn & Fels, 2015). Previous research shows a potential gap in investigating gamification to support communication practices, especially with close social ties.

There is concern about the communication habits of young adults in terms of their excessive use of smartphones and social media platforms. There is evidence, *e.g.*, of a relation between social media addiction and decreased mental health and academic performance (Hou et al., 2019). Some applications incorporate persuasive design principles that increase smartphone use and addictive behaviors (Chen, 2021). There is therefore a fine line between persuasive systems and digital addiction (Cemiloglu et al., 2021). The goal of the present work is to persuade university students who might be feeling disconnected from their support networks to keep in touch with them, but we acknowledge that this goal requires treading carefully since increasing smartphone use overall may be associated with negative effects such as smartphone and social media overuse or addiction.

## Understanding University Students’ Communication Preferences

In order to answer our first research question (*(1) How do university students in southern Chile communicate?*), we conducted a survey to understand university students’ communication preferences. In this section, we present the materials and methods, as well as the results obtained from this survey.

### Materials and methods

This section describes the students who participated in the study, as well as the instruments that were used to learn about student communication preferences. The study was approved by the Ethics Committee at Universidad Católica de la Santísima Concepción (code VRIP 08).

#### Participants

The number of computer science students at the university where this research was carried out is 280. To include a representative sample of this population, the recommended sample size was found to be 77 (Ryan, 2013). The questionnaires were answered in person on paper, and the survey was administered over two weeks. The participants were 90 students (15 women, 75 men) of Computer Science Engineering, which corresponds to 32% of the population. The average age of participants was 22(standard deviation = 3.00), and the age of the participants ranged from 18 to 30. All of them had a smartphone, with all but 18 phones using the Android operating system. The Android smartphones were of 11 different brands, mainly Samsung, Huawei and Motorola.

#### Methods

To study how students in a university in southern Chile communicate, we built a three-part survey: first, a basic demographic questionnaire; second, two questions about the phone each participant had; third, whether they use any of the following communication tools (or others): calls, SMS, Facebook, Facebook Messenger, Twitter, Instagram, WhatsApp, Snapchat, the frequency of use of each, and who they communicate with (relatives, parents, friends, significant other, classmates, others or unspecified). The survey form was analyzed for content validity through the expert judgment of three professionals (Corral, 2009).

### Results

Participants were asked to state the frequency of communication with each of their contact types, *i.e.,* how often they communicated with their friends through each medium. Therefore, participants may have responded *e.g.*, that they communicate through phone calls daily with their significant others, and weekly with their parents. Table 1 displays the number of students who interact with their social network, and the self-reported frequency of communication using each communication tool. Only the highest frequency is considered for each communication tool—*i.e.,* in the previous example, the participant would be considered as using phone calls daily (and not once a week).

**Table 1:** Communication tool and frequency (*N* = 90).

	**Phone calls**	**SMS**	**Facebook**	**Facebook messenger**	**Twitter**	**Instagram**	**WhatsApp**	**Snapchat**	**Other**
Daily	44	5	56	37	5	41	79	1	3
Every two days	19	1	7	12	2	1	0	0	5
Once or twice a week	16	7	3	13	2	6	2	0	2
Less than once a week/Never	11	77	24	28	81	42	9	89	80

WhatsApp is used by 87.8% (79/90) of participants daily and by 90.0% (81/90) at least once a week. Phone calls are used by 87.8% (79/90) of participants at least once a week, followed by Facebook (73.3%, 66/90), Facebook Messenger (68.9%, 62/90), and Instagram (53.3%, 48/90). Twitter, Snapchat, and SMSs, are used less often—they are never used, or used less than once a week, by 90.0% (81/90), 98.9% (89/90), and 85.6% (77/90) of participants respectively. Other communication tools, *e.g.*, Telegram and Tinder, were reported as used by very few participants (86.7%, 78/90, did not report using any other communication tool).

We also studied whether communication tools use differs when considering relationship, *i.e.,* whether some tools are used most often for some types of relationships than others. Overall, considering participants that stated they communicated at least once a week, 96.7% (87/90) of participants stated they communicated with their friends, 83.3% (75/90) with relatives and 65.6% (59/90) with parents. The rest of the categories all had less than 35% of respondents. Table 2 shows the communication tools participants use for each relationship type. In this case, if a participant *e.g.*, uses SMS and WhatsApp to communicate with their significant other, they will appear in both categories (so neither columns nor rows add up to the number of participants). Here, we can see that phone calls are the preferred medium when talking to parents, but WhatsApp is more often used to talk to relatives or friends.

**Table 2:** Communication tool and relationship.

	**Phone calls**	**SMS**	**Facebook**	**Facebook messenger**	**Twitter**	**Instagram**	**WhatsApp**	**Snapchat**	**Other**
Relatives	50	13	24	20	2	7	62	0	2
Parents	58	5	9	9	0	3	34	0	0
Friends	43	12	58	57	8	45	76	1	7
Sign. Other	20	8	6	5	0	4	17	1	0
Classmates	3	1	3	10	0	1	5	0	0
Other	5	1	13	6	8	10	7	0	5

### Discussion

The survey about communication preferences sought to understand university students’ communication preferences. The questionnaire was answered by 90 students of the Computer Science Engineering program.

The results of this survey are well aligned with previous research of social media use in Chile (*e.g.*, Poushter, Bishop & Chwe, 2018; Gómez-Urrutia & Tello-Navarro, 2020)—the most popular communication tools are WhatsApp and Facebook—although a large number of students also talk on the phone, which has generally been declining in popularity as a communication method when compared to text. This may be because texting is preferred to maintain constant contact, while calling offers a more personal and detailed interaction (Markham et al., 2017). We also found that, similarly to previous studies (*e.g.*, Russo et al., 2014), communication method depends on social tie: *e.g.*, the most frequent way to talk to their parents were phone calls, while the most frequent communication tool for friends was WhatsApp.

This survey has some limitations we would like to acknowledge. First, the participants of the study were only from one specific program at one specific university, and as such, results are not generalizable to other populations. It is important to note that the ratio of men to women respondents was 5 men per each woman. Furthermore, the data was self-reported, so it may incorporate biases and inaccuracies. Finally, the data is only quantitative and as such, cannot uncover motivations or explanations for why each communication method is used.

## Social+Me: persuasive application to motivate students’ communication

Based on the results described in the previous section, we developed a persuasive application called *Social+Me*. The application was implemented for Android version 4.0 (Ice Cream Sandwich) and higher, using Android Studio 3.1. This section describes the design of the *Social+Me* application and its main characteristics.

### Social+Me: Definition of Persuasive Strategies and Communication Tools

To develop the application, we started by defining the persuasive strategies, based on a recent survey of persuasive technologies for health and wellness (Orji & Moffatt, 2018), a review of existing applications available in the Google Play store (7 Weeks, 2019; Familyar, 2019; PhoneWatcher, 2019), and previous experience in the development of persuasive applications (Bascur et al., 2018). From this analysis, we chose six persuasive strategies to incorporate into our application. These strategies are: (1) *tracking/monitoring users*, (2) *social support and sharing*, (3) *the use of messages and reminders*, (4) *goals*, (5) *images*, and (6) *feedback in visual, audio and text form*.

We used the metaphor of a *tree in a field*, which we had used previously (Bascur et al., 2018; Rossel et al., 2019), to organize the persuasive strategies in an understandable and coherent way. A similar concept (using a garden) was used for Ubifit Garden (Consolvo et al., 2008), and similar ideas have been used previously with positive results (Oyebode, Maurya & Orji, 2020). In our case, the images in the metaphor change according to the progress of each user. This progress has a direct relation with the amount of communication that users establish through their mobile devices with their social network.

To define which communication tools would be included in the final persuasive application, we considered the survey presented in Section *Understanding University Students’ Communication Preferences*, which found that the most popular tools (considering weekly use) among university students in order of decreasing popularity were WhatsApp, phone calls, Facebook, Facebook Messenger, Instagram, SMS, Twitter and Snapchat (Table 3). Due to Facebook and Instagam API authorization limitations, we were not able to include them in the application, and since Snapchat only was used by 1.1% (1/90) of the users, it was not included as well. Therefore, the application includes the rest of the communication tools –*i.e.,* calls, WhatsApp, SMS and Twitter.

**Table 3:** Percentage of participants that use communication tools (descending order).

**Communication tool**	**Percentage of use**
Phone calls	96%
WhatsApp	90%
Facebook	82%
Facebook messenger	81%
Instagram	57%
SMS	26%
Twitter	18%
Snapchat	2%
Other	13%

### Social+Me: metaphor and social aspects

**Figure 2:**
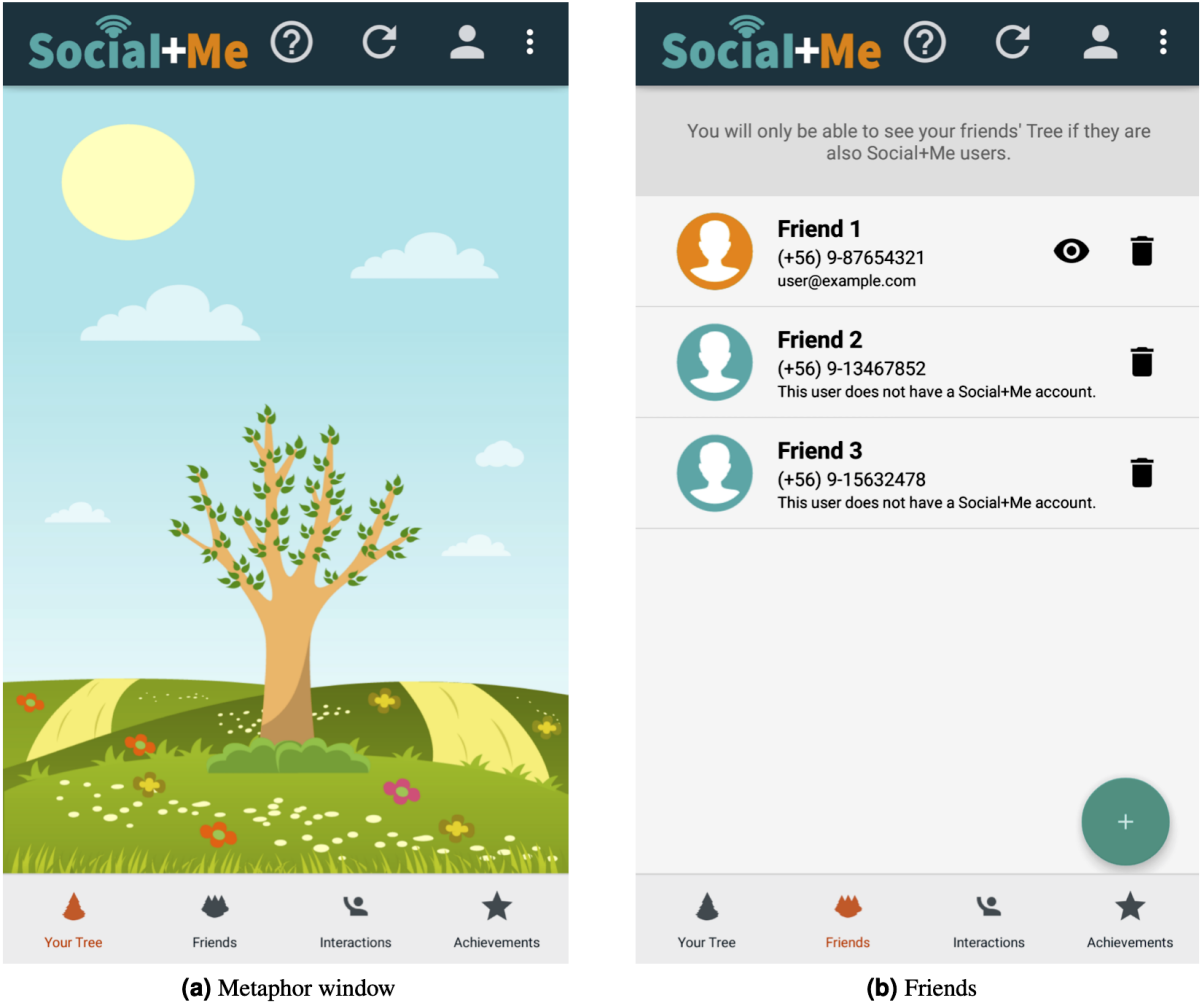
*Social+Me* final version.

The main element of the application is the metaphor of a *tree in a field* (Fig. 2). As the user interacts more with their social network (the *tracking/monitoring users* persuasive strategy), a greater number of elements are added to the tree (the *images* persuasive strategy).

To start using Social+Me, the user must grant the application permission to access Twitter and phone data, and then select their closest contacts, whose communication will be monitored by the app. Then, for each contact, additional information is requested (*e.g.*, Twitter handle). The selected contacts will appear in the “friends” tab, as shown in Fig. 2.

**Figure 3:**
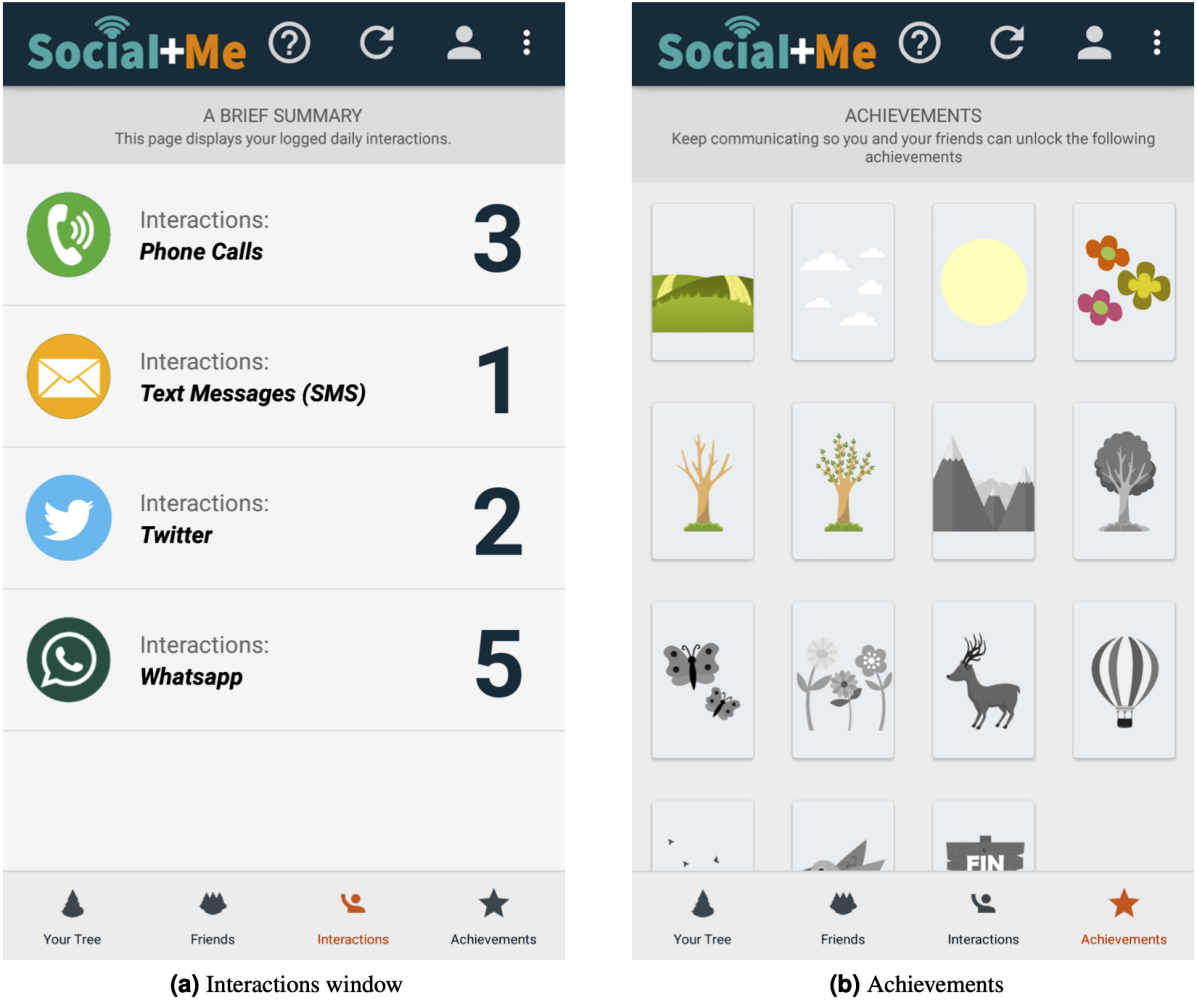
*Social+Me* final version (continuation).

The application provides information about the number of daily interactions the user had with their social network (the *feedback in visual, audio and text form* persuasive strategy), which is shown in Fig. 3. It also provides a social aspect, as if a friend has the Social+Me application, an eye-shaped icon appears next to their information, allowing the user to see the friend’s progress in the metaphor window view (the *social support and sharing* persuasive strategy).

The application provides 15 goals, or achievements, for users to reach (Fig. 3). These achievements are represented as parts of the landscape. As a user increases their communication with their social network, new elements are added (the *goals* persuasive strategy). Each new achievement is unlocked when the user interacts seven times in one day with users in their contact list. For instance, a user with 16 interactions in one day will earn two achievements (⌊16/7⌋ = 2). The next day, the interaction counter restarts, so they will need seven new interactions to earn their next achievement. If a user interacts 6 or less times in one day, they will lose one of their previously earned achievements.

### Notifications

**Figure 4:**
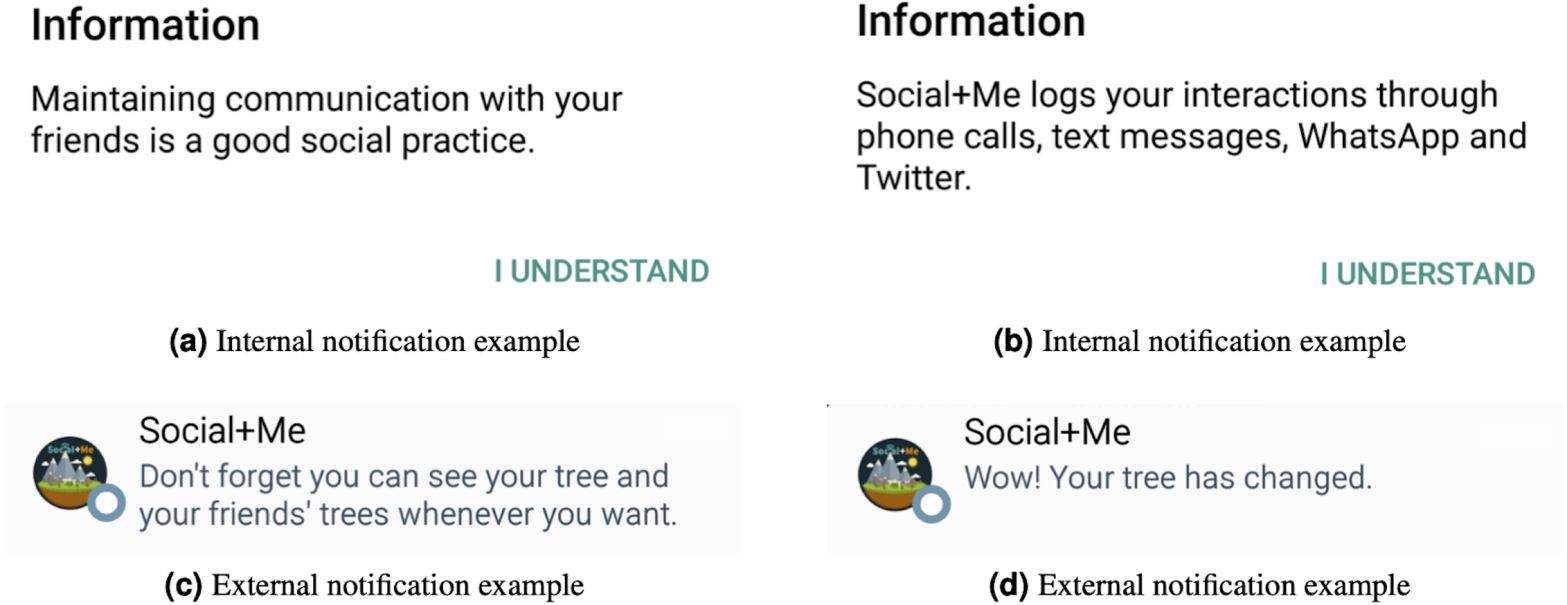
*Social+Me* final version (continuation).

The application implements non-diverse notifications (Kocielnik & Hsieh, 2017) for the users (the *the use of messages and reminders* persuasive strategy). Non-diverse notifications direct the attention of the users towards the metaphor, through simple and straightforward messages. The notifications were divided into two groups: internal and external. The internal notifications are those that are shown to users when they are using the application, and the external ones are those that are shown to users when the application is running as a background service. There are 13 different notifications and they are presented randomly to users. Two internal and two external notifications are shown in Fig. 4.

## Evaluation of Social+Me

In order to answer the second research question (*(2) Can a mobile application persuade university students to increase their communication patterns?*), we conducted an initial exploratory evaluation in which seven participants used the application for one week. This section describes the study participants and the methods used to evaluate the application and collect participant data.

### Materials and methods

This section describes the study participants, as well as the methods used to gather data during the evaluation. This study was approved by the Universidad Católica de la Santísima Concepción Ethics Committee (code VRIP 08).

#### Methods

At the beginning, each participant answered one questionnaire about digital skills, and then used the application for seven days with no intervention. After the seven days, the participants were visited by a researcher and filled out a usability questionnaire, verbally gave their opinion about the application, and completed an ad-hoc questionnaire about Social+Me application. Participants were not compensated for their participation.

The following instruments were used to collect participant data:

1. *DIGCOMP*: DIGCOMP is a test that is used to measure digital skills, and in which users are categorized into four possible levels of digital skills: *none*, *low*, *basic* or *above basic* (Ferrari, 2012).
2. *System Usability Scale (SUS)*: SUS was used to measure system usability (Brooke, 1996).
3. *Verbal questions*: Participants were asked two questions after their participation (“*Was the application useful for increasing digital communication with your social network, and if so, why?”*, and *“What positive or negative comment could you give about the application?*”). These opinions were recorded, and one researcher listened to the recordings and jotted down salient points that the participants made.
4. *Social+Me Satisfaction Questionnaire (SSQ)*: An ad-hoc questionnaire with a 5-point Likert scale was created to ask users their opinions about the application (see Table 4). The Chronbach-Alpha (Cronbach, 1951) value to measure internal consistency(reliability) of the questionnaire was 0.80 (good) (DeVon et al., 2007).

The study was carried out through the following steps.

1. Initially, one researcher gave a brief explanation about the purpose of the research and answered the participant’s questions. The participant then signed the consent form (10–15 min).
2. The participant answered the DIGCOMP questionnaire and a basic demographic questionnaire, *e.g.*, age, gender, educational level, occupation, brand and model of their smartphone (5–10 min).
3. The application was installed on the smartphone, and the researcher verified that it was working properly (10–15 min).
4. The researcher gave the participant a brief overview about the persuasive application and how to use it (15–20 min).
5. The participant used the application during seven days, with no researcher intervention.
6. The participant completed the SUS and SSQ questionnaires about Social+Me (5–10 min).
7. Finally, the researcher asked the two verbal questions to gather further information about users’ opinions (5–10 min).

#### Participants

We contacted seven participants through snowball sampling to participate in a first evaluation of the Social+Me application. The participants were all university students (the target users of our application). All of them were Engineering students and four of them had a computer science background. Six of the participants were male, and one was female. Their average age was 24 (standard deviation = 3.6). All participants had above basic digital skills—all of them had over 18/20 of the maximum score.

**Table 4:** Social+Me (SSQ) questionnaire.

Item	
1	The way to grant interaction permissions between Social+Me and Phone Calls, SMS, WhatsApp and Twitter was appropriate.
2	I enjoyed visualizing my level of digital communication through the metaphor of *tree in a field*.
3	The metaphor of *tree in a field* helped me to maintain or increase my digital communication with my social network.
4	The way to input contacts to my social network was simple.
5	It was simple to visualize the summary of the daily interactions recorded.
6	It was motivating for me to have a section of achievements to unlock.
7	It was easy to edit my user profile.
8	The tutorial shown when starting to use the application helped me understand how to use it.
9	The loading times of the different sections of the application (Metaphor, Friends, Interactions, Achievements, Profile, etc.) were adequate.
10	The application was available when I wanted to use it.

### Social+Me usability

The average SUS score given to the application was 85 (excellent) (Bangor, Kortum & Miller, 2009). All SUS scores were over 75, so all users considered that the usability was good (Lewis & Sauro, 2009). In the SSQ questionnaire, we obtained an average score of 4.5(out of 5).

Participants were asked to comment on positive and negative aspects about the application itself. They felt that Social+Me was easy and attractive to use, and that the tabs helped understand locked and unlocked achievements. One participant stated, *“The design of the *tree in a field* metaphor, the logo of the application, and the colours used were attractive to me”*.

### Social+Me persuasion

**Figure 5:**
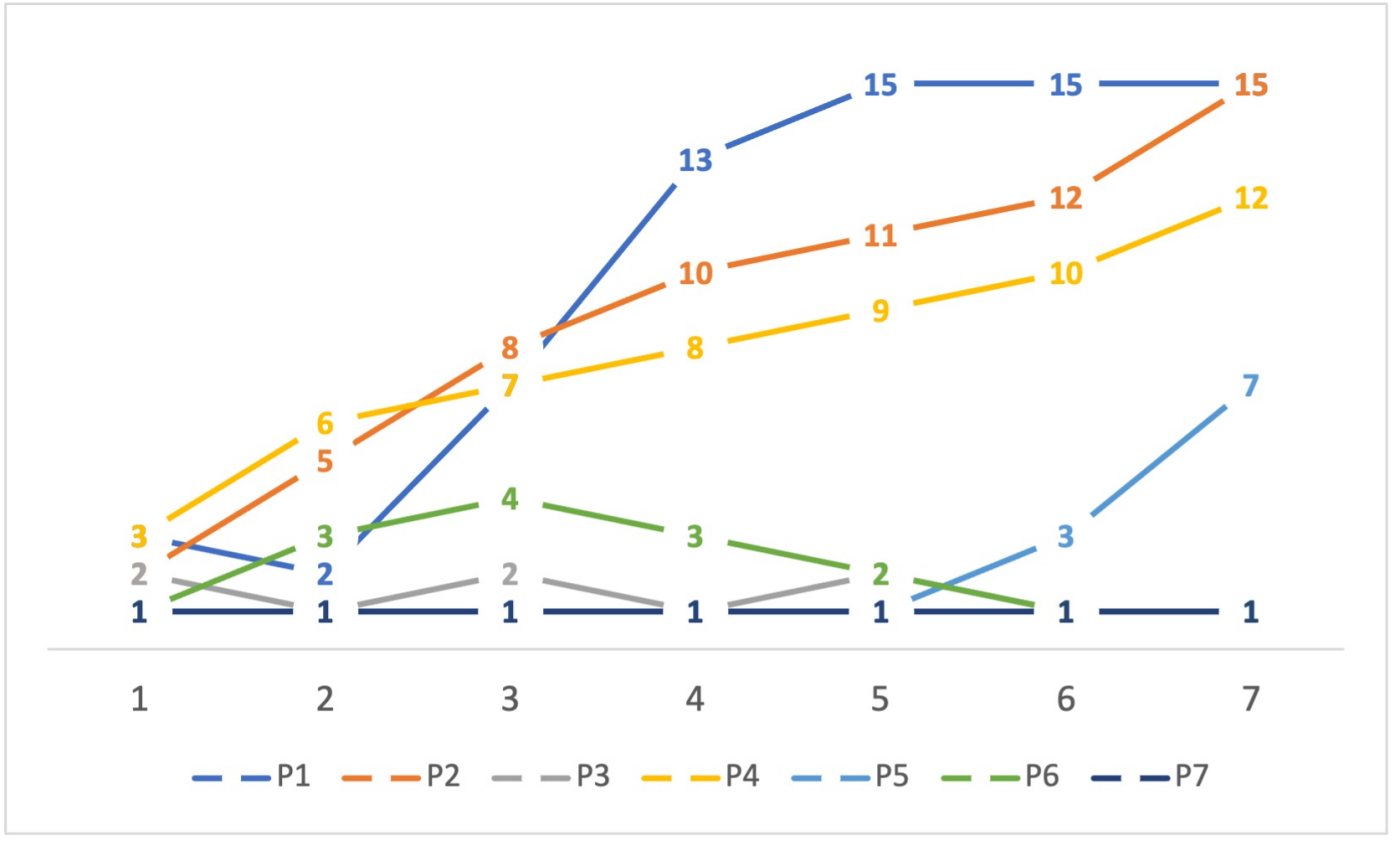
Achievements obtained by each participant, per day.

**Figure 6:**
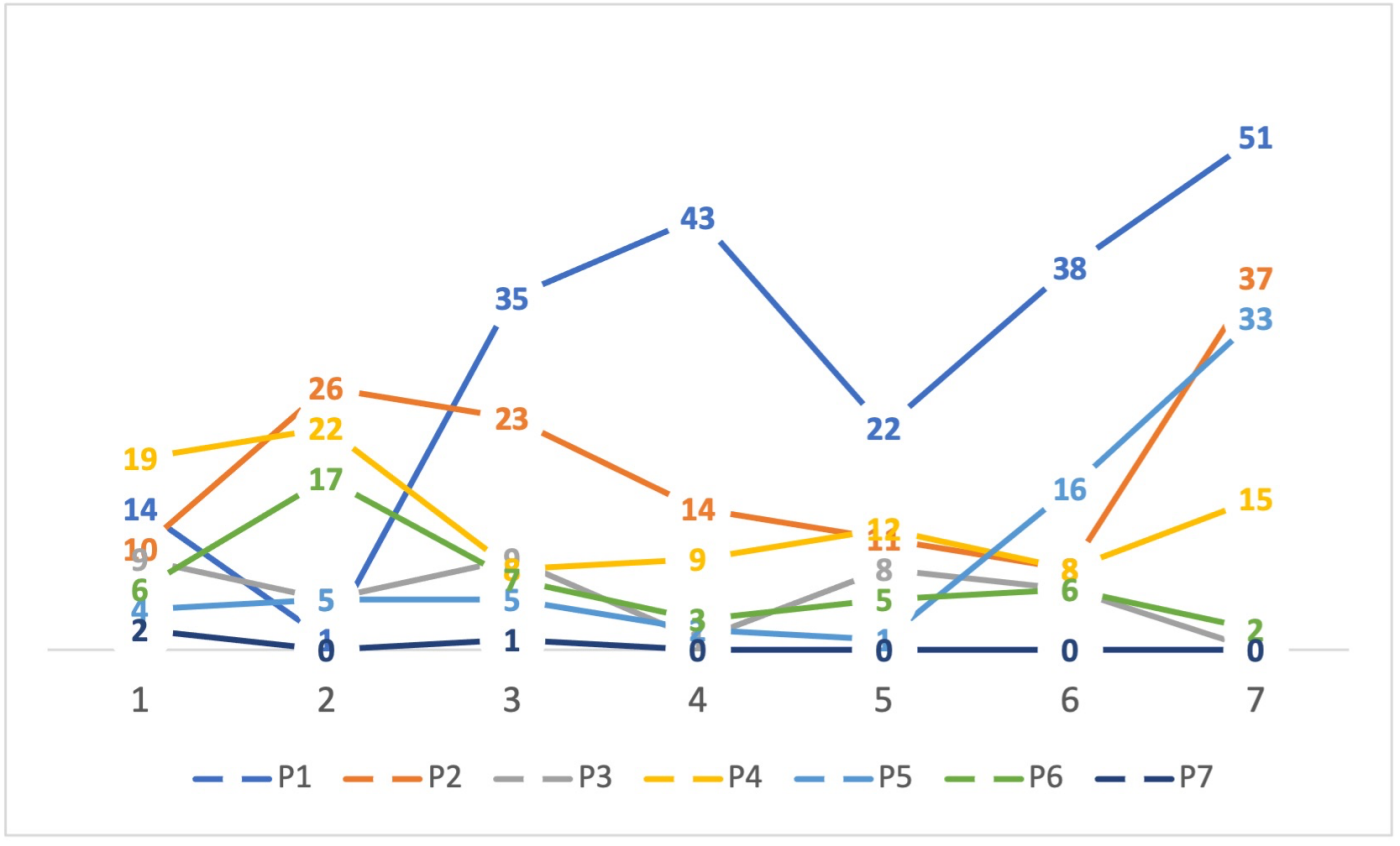
Number of interactions by each participant, per day.

The average number of recorded interactions was 11.8/day. Three of the participants obtained more than ten achievements in seven days, while three ended the seven days without achievements (either because they did not interact often enough or because they stopped interacting and lost their achievements). Figure 5 shows the achievements each participant obtained as well as their trajectory throughout the seven days. The number of interactions per day are shown in Fig. 6.

Participants were asked whether they felt that the application helped them increase their digital communication. Some users commented that Social+Me helped them increase their communication with their social network and recognize their communication levels. One participant said, *“For me, Social+Me was a daily communication challenge with my friends who are far away”*. Another one said, *“The application itself is good; the goal seems important and necessary for anyone who feels a lack of communication with their environment”*. Some participants stated that the persuasive aspects of the interface motivated them, *e.g.*, wanting to complete the landscape with their achievements. One participant stated *“It was very motivating to have the achievements section. I felt like it was a game, with me trying to unlock every achievement. That helped me stay connected”*.

### Discussion

The design of the Social+Me application aims to promote sustained communication practices between young adults and their closest networks (family members and friends) using technology, providing young adults support and reducing isolation. The application was found to have good usability. Some users also found the metaphor engaging, and they felt that the application persuaded them to maintain communication.

The participants in this initial evaluation were only seven, and the application was only used for seven days, so it is not possible to extrapolate any findings to larger populations or periods of time. However, it is interesting to note that two participants increased their communication during the period (P1, P5), and five maintained their communication within their initial range, with certain fluctuations. Of those participants, four started at a medium or low level of interaction (P2, P3, P4, P6) and one started at a very low level of interaction (P7). Since we did not have a previous period with no intervention to compare with, we cannot be certain whether the application affected communication levels, *e.g.*, it is possible that those participants with steady medium levels of interaction had previously much higher or lower interaction levels. If we analyze Fig. 5, we can see that participants P2 and P4 did have a steady progress towards gaining achievements. When combined with the participant comments, we can infer that the application did persuade some of the participants to increase their communication, though naturally, not all of them, since we did not measure whether participants were receptive to the idea of increasing their communication, as is suggested in these types of studies (Fogg, 2009). If this application was to be tested for a longer period and a wider audience, some design decisions should be reconsidered, *e.g.*, the number of interactions to earn achievements, which was defined arbitrarily as seven and should be tailored to previous activity levels. For example, for participant P7, who seems to interact infrequently, the threshold to begin gaining achievements should be lower, while for participant P1, who earned all 15 achievements by the fifth day, the threshold should be higher or more achievements should be available.

One of the goals of the Social+Me application was to decrease isolation—which can affect students as well as affect their learning at university (Lim & Vighnarajah, 2018)—and persuade users to keep in touch with their support networks. The use of persuasive mechanisms has been applied in online social networks for many years (Weiksner, Fogg & Liu, 2008), for several purposes, *e.g.*, marketing (Weiger, Hammerschmidt & Wetzel, 2018). However, there are also persuasive design characteristics inserted into social media itself to entice users to keep using it, and these do not consider user objectives, time constraints, relationships and mental wellness (Bedjaoui, Elouali & Benslimane, 2018). Our study is innovative in that it aims to increase the use of smartphone communication to decrease isolation—*i.e.,* to purposefully engage students with their support networks—and that the participants are aware of this goal.

Although the goal itself may be positive, the persuasive use of technology may be perceived as a problematic or addictive (Lopez-Fernandez et al., 2017; Panova & Carbonell, 2018). From the beginning of their development, researchers have discussed the ethics of persuasive technology; similarly, now the interactive design community has been focus of criticism for its role in the emergence of dark patterns (Rogers et al., 2021), or subtle interface tricks that make users do things they do not want to do. One straightforward “golden” rule of persuasive technology is that it should not persuade someone of something the creators would not want to be persuaded of themselves (Berdichevsky & Neuenschwander, 1999). Similarly, others have stated that persuasive applications are ethical if they influence others of behavior that users would have held as goals themselves (Timmer, Kool & van Est, 2015). However, in 2009, a review of papers published in a persuasive systems conference found that ethics were often unaddressed in research studies (Torning & Oinas-Kukkonen, 2009). We would like to acknowledge that our study does present ethical issues. While our goal is to encourage students to keep in touch with their networks, excessive social media use and fear of missing out are related to serious issues such as smartphone addiction (Gezgin, 2018), so further work should be careful to balance improving communication and preventing excessive smartphone use.

This study has several limitations we would like to acknowledge. First, the study was only carried out for a short period of time and did not compare previous communication patterns to those while using the application. Second, the study had only seven participants, and they were not necessarily interested in improving their communication patterns nor suffering from isolation. Furthermore, the participants may have had positive biases, since they were recruited through snowball sampling from one researcher. For these reasons, the study is preliminary and although the results are promising, we are not able to make claims about longer term issues such as persuasion, adoption, or excessive use of the application. We would also like to conduct more in-depth qualitative evaluation to further understand the motivations behind the use of the application, as well as a longer term quantitative study that allows us to study the issues of persuasion and overuse with more depth. We recognize as well self-report limitations, particularly the potential influence of *Social Desirability Bias* (SDB); although we used validated surveys for our study (SUS, Digital skills and SSQ) at this preliminary evaluation of Social+Me we did not include SDB mitigation techniques (Kwak, Ma & Kim, 2021; Durmaz, Dursun & Kabadayi, 2020).

## Conclusions

This paper presented a study of communication preferences in a southern Chilean university students as well as Social+Me, a persuasive application to promote university students’ communication practices with their support networks. Our study found that the most popular tools for communication were phone calls and WhatsApp, used to communicate with friends, relatives, and parents. The Social+Me application monitors users’ communication with their support networks, presenting a metaphor of a *tree in a field* populated with positive elements (*e.g.*, leaves, clouds, flowers, animals) as communication increases. The novel use of persuasive elements to increase communication with the aim of reducing isolation should be implemented and monitored carefully as not to increase social media or smartphone addiction, therefore increasing isolation.

Social+Me aims to promote sustained communication practices between young adults and their closest networks. Our findings, although preliminary, are promising. Users found Social+Me to have good usability, an engaging metaphor, and they felt persuaded to maintain communication. We identified design decisions that should improve, *e.g.*, the number of interactions to earn achievements should be dynamically assigned accordingly to the user’s activity level. The goal of increasing smartphone communication to decrease isolation requires further study, to find a balance between communication and preventing excessive smartphone use that might lead to smartphone addiction.

Future work to gather accurate data about communication practices should objectively measure communication practices between university students and their social ties, *e.g.*, through retrospective logging. Future work to improve the Social+Me application should consider integrating more communication platforms, as well as personalizing achievement thresholds. Finally, we suggest that it may be important to explore, in future work, contextual-cultural variables that may impact communication practices.

## Supplemental Information

10.7717/peerj-cs.848/supp-1 Supplemental Information 1 University students’ communication preferences and social networks interactions through Social+Me

10.7717/peerj-cs.848/supp-2 Supplemental Information 2 Communication preferences questionnaire (English version)

10.7717/peerj-cs.848/supp-3 Supplemental Information 3 Communication preferences questionnaire (Spanish version)

## Additional Information and Declarations

### Data Availability

The following information was supplied regarding data availability: The raw data are available in the Supplementary File.
