# Supplementary material for: Social+Me: a persuasive application to increase communication between students and their support networks in Southern Chile
Source: PeerJ Comput Sci. 2022 Jan 27;8:e848. doi: 10.7717/peerj-cs.848 (PMC8802782; doi:10.7717/peerj-cs.848)
Supplement: Supplemental Information 2 [file peerj-cs-08-848-s002.pdf]

Survey on communication preferences

Fill the following fields with your personal information.

Age: ____

Gender:
____ Female.
____ Male.

Educational level:
____ Incomplete Primary Education.
____ Complete Primary Education.
____ Incomplete Secondary Education.
____ Complete Secondary Education.
____ Incomplete Technical Education.
____ Complete Technical Education.
____ Incomplete University Education.
____ Incomplete University Education.

What is your current profession or activity?

Do you own a smartphone? :
____ Yes.
____ No.

If the answer above is “Yes”, what brand and model of smartphone do you have?

For the following types of digital communication, put an "X" in the corresponding line.

1. Phone Calls:

Do you use them? Yes ____ No ____

If your answer is "Yes", please answer the following question:

Frequency of use	With whom? (Kinship/Relationship)
___ Daily.	
___ Every two days.	
___ Once or twice a week.	
___ Less than once a week.	

2. Texting (Short Message Service, SMS):

Do you use them? Yes ____ No ____

If your answer is "Yes", please answer the following question:

Frequency of use	With whom? (Kinship/Relationship)
___ Daily.	
___ Every two days.	
___ Once or twice a week.	
___ Less than once a week.	

3. Facebook.

Do you use it? Yes ____ No ____

If your answer is "Yes", please answer the following question:

Frequency of use	With whom? (Kinship/Relationship)
___ Daily.	
___ Every two days.	
___ Once or twice a week.	
___ Less than once a week.	

4. Facebook Messenger.

Do you use it? Yes ____ No ____

If your answer is "Yes", please answer the following question:

Frequency of use	With whom? (Kinship/Relationship)
___ Daily.	
___ Every two days.	
___ Once or twice a week.	
___ Less than once a week.	

5. Twitter.

Do you use it? Yes ____ No ____

If your answer is “Yes”, please answer the following question:

Frequency of use	With whom? (Kinship/Relationship)
___ Daily.	
___ Every two days.	
___ Once or twice a week.	
___ Less than once a week.	

6. **Instagram.**

Do you use it? Yes ____ No ____

If your answer is “Yes”, please answer the following question:

Frequency of use	With whom? (Kinship/Relationship)
___ Daily.	
___ Every two days.	
___ Once or twice a week.	
___ Less than once a week.	

7. Whatsapp.

Do you use it? Yes ____ No ____

If your answer is "Yes", please answer the following question:

Frequency of use	With whom? (Kinship/Relationship)
___ Daily.	
___ Every two days.	
___ Once or twice a week.	
___ Less than once a week.	

8. Snapchat.

Do you use it? Yes ____ No ____

If your answer is "Yes", please answer the following question:

Frequency of use	With whom? (Kinship/Relationship)
___ Daily.	
___ Every two days.	
___ Once or twice a week.	
___ Less than once a week.	

9. Others.

Which? ____________________

Do you use it? Yes ____ No ____

If your answer is "Yes", please answer the following question:

Frequency of use	With whom? (Kinship/Relationship)
___ Daily.	
___ Every two days.	
___ Once or twice a week.	
___ Less than once a week.	
