# Supplementary material for: Social+Me: a persuasive application to increase communication between students and their support networks in Southern Chile
Source: PeerJ Comput Sci. 2022 Jan 27;8:e848. doi: 10.7717/peerj-cs.848 (PMC8802782; doi:10.7717/peerj-cs.848)
Supplement: Supplemental Information 3 [file peerj-cs-08-848-s003.pdf]

Encuesta sobre preferencias de comunicación

Complete los siguientes campos respecto a su información personal.

Edad: ____

Sexo:
____ Femenino.
____ Masculino.

Nivel Educacional:
____ Básica Incompleta.
____ Básica Completa.
____ Media Incompleta.
____ Media Completa.
____ Técnica Incompleta.
____ Técnica Completa.
____ Universitaria Incompleta.
____ Universitaria Completa.

¿Cuál es la profesión o actividad ejerce actualmente?

¿Posee un celular tipo Smartphone? :
____ Sí.
____ No.

Si su respuesta anterior es "Sí", ¿Qué marca y modelo de celular posee?

Para los siguientes tipos de comunicación digital, marque con una "X" la casilla correspondiente.

1. Llamadas telefónicas:

¿Lo utilizas? Sí ____ No ____

Si su respuesta es "Sí" responder a la siguiente pregunta:

Frecuencia de uso	¿Con quién? (Parentesco / Relación)
___ Diaria.	
___ Cada dos días.	
___ 1-2 vez por semana.	
___ Menos de 1 vez por semana.	

2. Mensajes de texto:

¿Lo utilizas? Sí ____ No ____

Si su respuesta es "Sí" responder a la siguiente pregunta:

Frecuencia de uso	¿Con quién? (Parentesco / Relación)
___ Diaria.	
___ Cada dos días.	
___ 1-2 vez por semana.	
___ Menos de 1 vez por semana.	

3. Facebook.

¿Lo utilizas? Sí ____ No ____

Si su respuesta es "Sí" responder a la siguiente pregunta:

Frecuencia de uso	¿Con quién? (Parentesco / Relación)
___ Diaria.	
___ Cada dos días.	
___ 1-2 vez por semana.	
___ Menos de 1 vez por semana.	

4. Facebook Messenger.

¿Lo utilizas? Sí ____ No ____

Si su respuesta es "Sí" responder a la siguiente pregunta:

Frecuencia de uso	¿Con quién? (Parentesco / Relación)
___ Diaria.	
___ Cada dos días.	
___ 1-2 vez por semana.	
___ Menos de 1 vez por semana.	

5. Twitter.

¿Lo utilizas? Sí ____ No ____

Si su respuesta es "Sí" responder a la siguiente pregunta:

Frecuencia de uso	¿Con quién? (Parentesco / Relación)
___ Diaria.	
___ Cada dos días.	
___ 1-2 vez por semana.	
___ Menos de 1 vez por semana.	

6. **Instagram.**

¿Lo utilizas? Sí ____ No ____

Si su respuesta es "Sí" responder a la siguiente pregunta:

Frecuencia de uso	¿Con quién? (Parentesco / Relación)
___ Diaria.	
___ Cada dos días.	
___ 1-2 vez por semana.	
___ Menos de 1 vez por semana.	

7. Whatsapp.

¿Lo utilizas? Sí ____ No ____

Si su respuesta es "Sí" responder a la siguiente pregunta:

Frecuencia de uso	¿Con quién? (Parentesco / Relación)
___ Diaria.	
___ Cada dos días.	
___ 1-2 vez por semana.	
___ Menos de 1 vez por semana.	

8. Snapchat.

¿Lo utilizas? Sí ____ No ____

Si su respuesta es "Sí" responder a la siguiente pregunta:

Frecuencia de uso	¿Con quién? (Parentesco / Relación)
___ Diaria.	
___ Cada dos días.	
___ 1-2 vez por semana.	
___ Menos de 1 vez por semana.	

9. Otros.

¿Cuál? ____________________

¿Lo utilizas? Sí ____ No ____

Si su respuesta es "Sí" responder a la siguiente pregunta:

Frecuencia de uso	¿Con quién? (Parentesco / Relación)
___ Diaria.	
___ Cada dos días.	
___ 1-2 vez por semana.	
___ Menos de 1 vez por semana.	
